# SNM1B/Apollo in the DNA damage response and telomere maintenance

**DOI:** 10.18632/oncotarget.16864

**Published:** 2017-04-05

**Authors:** Maren Schmiester, Ilja Demuth

**Affiliations:** ^1^ Lipid Clinic at the Interdisciplinary Metabolism Center, Charité-Universitätsmedizin Berlin, 13353 Berlin, Germany; ^2^ Institute of Medical and Human Genetics, Charité–Universitätsmedizin Berlin, 13353 Berlin, Germany; ^3^ Research Group on Geriatrics, Charité–Universitätsmedizin Berlin, 13347 Berlin, Germany

**Keywords:** Apollo, hSNM1B, DCLRE1B, Fanconi anemia

## Abstract

hSNM1B/Apollo is a member of the highly conserved β-CASP subgroup within the MBL superfamily of proteins. It interacts with several DNA repair proteins and functions within the Fanconi anemia pathway in response to DNA interstrand crosslinks. As a shelterin accessory protein, hSNM1B/Apollo is also vital for the generation and maintenance of telomeric overhangs. In this review, we will summarize studies on hSNM1B/Apollo's function, including its contribution to DNA damage signaling, replication fork maintenance, control of topological stress and telomere protection. Furthermore, we will highlight recent studies illustrating hSNM1B/Apollo's putative role in human disease.

## INTRODUCTION

Interstrand crosslinks (ICLs) are amongst the most cytotoxic DNA lesions, covalently linking both DNA strands and inhibiting transcription and replication of the cell's genetic material. Naturally occurring endogenous (e.g. acetaldehyde, a metabolite of the glycolytic pathway) and exogenous agents (e.g. psoralen + ultraviolet A light, “PUVA”) are capable of inducing ICLs. Additionally, some compounds widely used in anti-cancer therapies, such as mitomycin C (MMC), cyclophosphamide and cisplatin owe their cytotoxic qualities to the generation of ICLs.

The rare genetic disease Fanconi anemia (FA) emphasizes the importance of adequate cellular mechanisms to tolerate and repair these lesions: biallelic mutations in one of the 20 known autosomal or a mutation in the single known X-chromosomal FA gene result in the accumulation of ICLs leading to bone marrow failure and an increased risk for cancer in affected individuals (reviewed e.g. in [1–4]). Processing of ICLs in mammalian cells is highly complex and relies on the intricate cooperation of several DNA repair pathways, including the FA pathway, nucleotide excision repair (NER), translesion synthesis (TLS), homologous recombination (HR) and non-homologous end-joining (NHEJ). In short, an ICL is recognized and incisions are made 3′ and 5′ of the lesion. If this “unhooking” takes place during DNA replication, a double-strand break (DSB) is induced; in the absence of replication, it results in a gapped intermediate. These DNA structures are then subject to further processing by nucleases, repair via HR or TLS and finally removal of the unhooked oligonucleotide (reviewed e.g. in [5–9]).

Investigation of the ICL repair mechanisms in lower organisms contributed significantly to our knowledge in this field. Beginning in the early 1980’s, several *Saccharomyces cerevisiae* mutants with specific hypersensitivitiy to ICL inducing agents were identified. Some of these *pso* (sensitive to *pso*ralen + UVA) and *snm* mutants (*s*ensitive to *n*itrogen *m*ustard) were later found to be allelic and the nomenclature *PSO1* through *PSO10* was introduced (reviewed e.g. in [10]). *PSO2* encodes a protein of 76 kDa (Pso2p) that is essential for the repair of DSBs resulting from ICL repair in replicating yeast and is believed to contribute to ICL repair in G1 and G2 phase as well. Pso2p is a 5′ exonuclease and has site-specific endonuclease activity for the opening of DNA hairpins. It has been suggested that Pso2p processes unhooked ICLs as well as DNA hairpins generated by ICL damage in order to provide substrates for downstream repair steps. Both the exonuclease and the endonuclease activity of Pso2p depend on an active site composed by its metallo-β-lactamase (MBL) domain (named after the structurally related MBL domain of prokaryotic enzymes hydrolyzing ß-lactam antibiotics) and the self-defining β-CASP domain (named after its representative members *C*PSF, *A*RTEMIS, *S*NM1, *P*SO2), which is found in a subgroup of proteins within the MBL-superfamily [11–14] (reviewed e.g. in [10, 15]). In mammalian cells, three proteins with closer sequence similarities to Pso2p have been identified: SNM1A, SNM1B/Apollo and SNM1C/Artemis (human gene symbols: DCLRE1A, DCLRE1B and DCLRE1C). All three proteins are involved in DNA processing and cell cycle regulation.

The first mammalian Pso2p homolog identified was the human hSNM1A (KIAA0086) protein. It has a protein sequence similarity of up to 48% to yeast Pso2p, the highest amongst all known homologs [16, 17], and encodes a 5′ exonuclease [18]. SNM1A is involved in the repair of ICLs and disruption of *SNM1A* leads to increased sensitivity towards ICL-inducing agents in chicken, mouse and human cells (reviewed e.g. in [10, 15]). Recently, it was shown that hSNM1A is able to digest DNA past interstrand crosslinks [19, 20].

Another *PSO2* homolog, *hSNM1C*, is mutated in patients with radiosensitive severe combined immunodeficiency (RS-SCID). In reference to the Hellenic goddess for the protection of children, this protein was named “Artemis” [21]. SNM1C/Artemis is involved in V(D)J recombination, a defining feature of the adaptive immune system. In response to DSBs, SNM1C/Artemis is phosphorylated by and complexes with DNA-PKcs and acquires endonuclease activity, cleaving 5′ and 3′ overhangs, flaps, gaps and hairpin structures [22, 23]. Hairpin opening is required for the processing of intermediates during V(D)J recombination. MEFs and DT40 cells deficient for SNM1C displayed increased sensitivities towards ionizing radiation, but not to ICL-inducing agents, indicating that the encoded protein is unlikely to play a major role in ICL repair (reviewed e.g. in [10, 15]).

hSNM1B/Apollo harbors a N-terminal amino acid sequence with 33% homology to Pso2p. The gene contains four exons, is located on chromosome 1p13.1–13.3 and encodes an open reading frame of 532 amino acids. An isoform of the transcript lacking exon 2 which could lead to the translation of proteins lacking the Pso2p homology domain was also detected, although the significance of this alternative-splicing product is unclear [24]. Consistent with the DNA processing functions of the SNM1 family proteins, hSNM1B/Apollo was found to be a DNA 5′ exonuclease with a preference for single-stranded substrates [20, 25]. Like Pso2p and its other homologs, endogenous hSNM1B/Apollo is expressed at very low levels, making its detection difficult [24, 26]. Current knowledge of hSNM1B/Apollo's functions suggests that the nuclease is essential for two major cellular processes: DNA damage response and telomere maintenance. In this article, we will review the current literature on SNM1B/Apollo in detail, focusing on the dual function of the protein and discussing its role in human disease.

### hSNM1B/Apollo's role in the DNA damage response

#### hSNM1B/Apollo is required for the normal cellular response to DNA interstrand crosslinks

In DT40 cells, a lack of SNM1B/Apollo results in an increase in sensitivity towards MMC and cisplatin [27, 28]. Depletion of hSNM1B/Apollo renders human cells hypersensitive towards ICL-inducing agents, resulting in reduced survival rates after treatment with MMC and cisplatin. As expected for mutants defective in ICL repair, hSNM1B/Apollo-depleted cells also show an increase in chromosomal aberrations upon exposure to these DNA crosslinkers. Interestingly, while results from our laboratory also suggest hypersensitivities towards IR in hSNM1B/Apollo-depleted HeLa cells, Bae and colleagues found no increased sensitivity after radiation of depleted HEK293 cells [24, 29]. Irradiation of GM00637 cells does not lead to an increase in hSNM1B/Apollo-foci positive cells in immunofluorescence experiments; however, the number of foci per nucleus increases significantly. This finding could be attributed to the low expression level of hSNM1B/Apollo, which might only cross the threshold for detection in a fraction of cells. Taken together, hSNM1B/Apollo functions in the response to ICLs and may also be involved in the response to IR-damaged DNA [24, 29–31].

### hSNM1B/Apollo is involved in both ATM- and ATR-mediated DNA damage signaling

hSNM1B/Apollo-deficient cells display defects in various cell cycle checkpoints. hSNM1B/Apollo depleted GM00637 cells permit release from the G2/M checkpoint despite IR-induced DNA damage and depleted HeLa cells do not decrease DNA synthesis in response to MMC exposure, indicating a defective S phase checkpoint [29, 31] Additionally, a prophase checkpoint defect was noted in these HeLa cells, which was attributed to the lack of interaction between hSNM1B/Apollo and the microtubule binding protein astrin [32]. Similar phenotypes have been observed in FA cells [33, 34].

Consistent with these observed cell cycle defects, markers of checkpoint activation are affected by hSNM1B/Apollo depletion. In response to ICLs, the ATR-mediated checkpoint is activated and repair is induced via the phosphorylation of specific substrates such as CHK1. Two other major protein kinases involved in DNA damage signaling, ATM and its effector kinase CHK2, are not known to play a substantial role in ICL-repair and are typically activated when DSBs are detected (reviewed e.g. in [35–37]). Interestingly, hSNM1B/Apollo-deficient HEK293 cells are defective in the phosphorylation of CHK2 in response to MMC, while CHK1 activation is unaffected [29]. Similar observations were made in other human fibroblasts, showing no increase in CHK1 phosphorylation after replication stress induction [38]. In contrast, CHK1 phosphorylation was found to be disturbed in hSNM1B/Apollo-depleted GM00637 cells following UVC exposure [39]. The phosphorylation of ATM proved to be reduced in depleted HEK293 cells, consistent with results from depleted GM00637 cells after IR showing a reduction in the phosphorylation of ATM and its substrates p53, H2A.X and SMC [31, 40]. hSNM1B/Apollo was shown to localize to sites of DNA damage induced by laser micro-irradiation independently of ATM, pointing to a role for the protein in the early stages of the DNA damage response [31].

As a whole, these findings suggest that hSNM1B/Apollo is involved in ATM- and perhaps also in ATR-mediated signaling after DNA damage, possibly by facilitating ATR's activation after the detection of an ICL and subsequently by allowing for ATM's activation during the repair-associated induction of a DSB. However, although ATM and ATR vary in their DNA damage specificities, they are to some extent redundant and are known to cross talk. Further research regarding the impact of hSNM1B/Apollo on the activation of ATM, and particularly ATR, is needed to decipher the exact role of the nuclease in this interplay.

### Differences in structure may account for hSNM1A's and hSNM1B/Apollo's specific nuclease activities

As mentioned above, hSNM1A can digest past ICL lesions [19, 20]. A current model of ICL repair suggests that this ability allows hSNM1A to processes the residual cross-linked oligonucleotide after unhooking of the lesion, leaving a single nucleotide covalently bound to the sister strand and providing a suitable substrate for further repair via TLS [41] (reviewed e.g. in [8]). Interestingly, hSNM1B/Apollo was shown to digest past ICLs as well, although its capacity to do so is lower than that of hSNM1A, at least with the type of ICL-substrate tested [20]. Nevertheless, hSNM1B/Apollo might be somewhat redundant to hSNM1A in ICL trimming and further studies are needed to elucidate their respective roles in this aspect of ICL repair. Recently, Allerston and colleagues reported the crystal structures of hSNM1A and hSNM1B/Apollo [42]. They found the overall architecture of their active sites to be similar, but discovered significant differences regarding the charge distribution surrounding the active sites. hSNM1A possesses a pronounced area of positive potential, which may limit the dislocation of DNA during processing and could explain hSNM1A's increased processivity on high molecular weight DNA. They also identified a putative DNA-binding groove in both proteins that is important for their processivity and ability to process cross-linked DNA. Differences in the structure of hSNM1A and hSNM1B/Apollo are therefore likely responsible for their specific roles in the DNA damage response.

### hSNM1B/Apollo is linked to the FA pathway

As discussed above, hSNM1B/Apollo depletion in human cells leads to hypersensitivity towards ICL-inducing agents, increased sensitivity towards IR, defects in various cell cycle checkpoints and chromosomal instability. These phenotypes are also hallmarks of cells derived from FA patients, raising the possibility that hSNM1B/Apollo acts within the FA pathway. The main function of the FA pathway is to orchestrate proteins involved in the repair of ICLs. Its molecular mechanisms can be divided into three steps: First, the so-called “upstream” FA proteins assemble into the FA core complex that is recruited to sites of DNA damage. Together with associated proteins, this complex catalyzes the second step of the pathway: the monoubiquitination of FANCD2 and FANCI. This complex in turn localizes to chromatin where it recruits and coordinates the activity of numerous downstream DNA repair proteins in the third step of the FA pathway (reviewed e.g. in [3, 43–46]).

**Figure 1:**
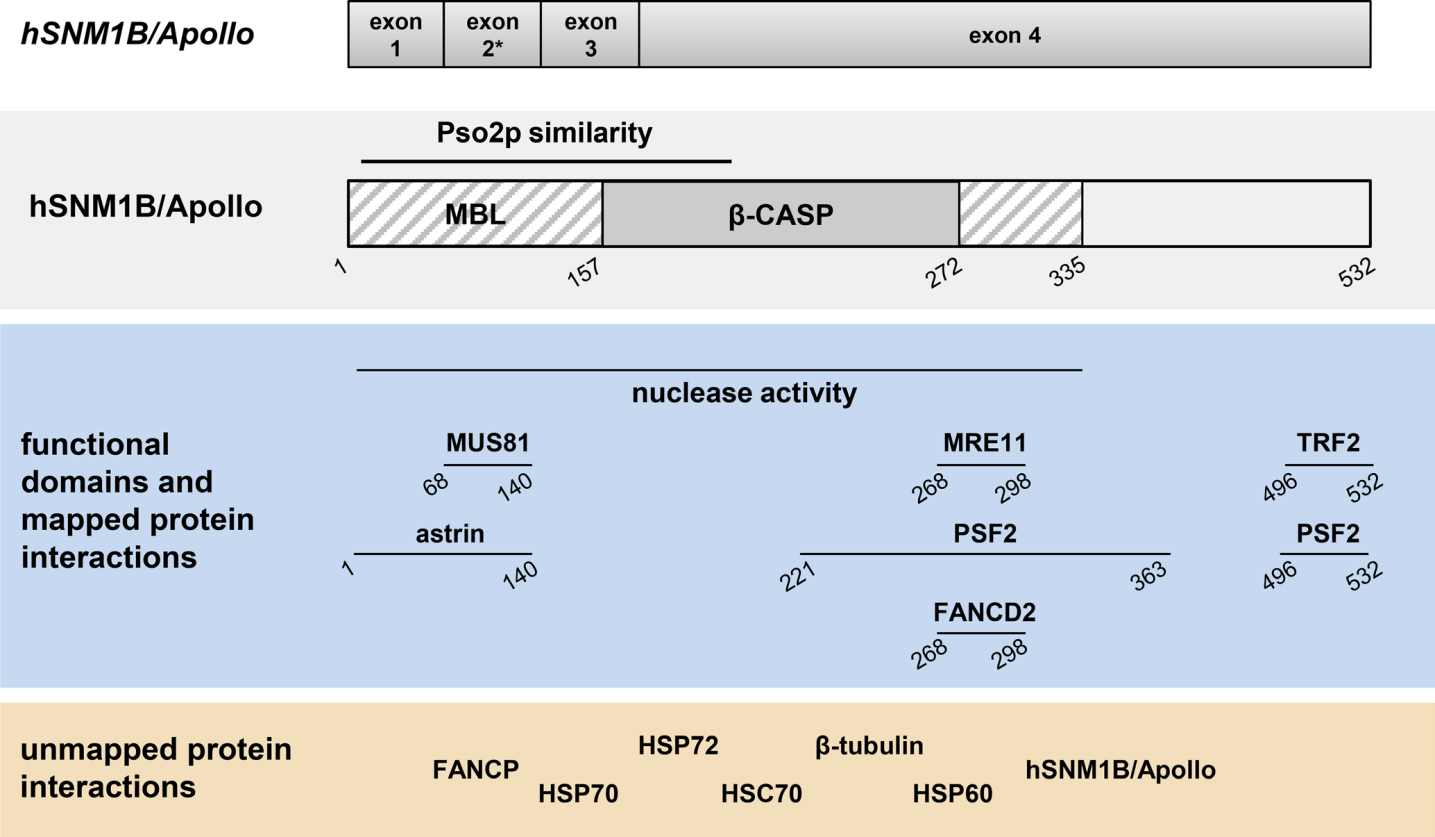
Schematic representation of the genomic organization, structural and functional domains and interacting partners of hSNM1B/Apollo Genomic organization and domains are described in the text. The asterisk indicates differential splicing of exon 2. Horizontal lines indicate identified domains or regions of interaction with the respective proteins. Numbers indicate amino acid positions. Pso2p: Pso2 protein; MBL: Metallo-β-lactamase; β-CASP: β-CPSF–Artemis–SNM1–PSO2.

hSNM1B/Apollo was found to interact physically with two FA proteins, FANCD2 and FANCP/SLX4. The FANCD2 interaction with hSNM1B/Apollo was mapped to the C-terminal end of the β-CASP domain (Figure 1) and is probably indirect. FANCD2 monoubiquitination is not affected by hSNM1B/Apollo depletion, suggesting that the nuclease acts downstream of the FA core complex [24, 29]. However, hSNM1B/Apollo is required for the effective assembly of FANCD2 into DNA repair foci [30, 38]. We initially reported that FANCP/SLX4 binds to hSNM1B/Apollo's N-terminus [47], however, recent results from our laboratory indicate that the GFP-tag fused to the N-terminal hSNM1B/Apollo fragment used in the earlier experiments distorted the interaction and that FANCP/SLX4 in fact interacts with at least two regions of hSNM1B/Apollo (Schmiester and Demuth, unpublished). hSNM1B/Apollo functions epistatically with FANCD2 and FANCP/SLX4 in the cellular response to ICL-induced DNA damage as shown by similar survival rates of single- and double-knockdown cells [30, 47]. Additionally, hSNM1B/Apollo and FANCD2 or FANCI respectively act in epistasis to suppress ICL-induced chromosomal aberrations. Furthermore, hSNM1B/Apollo deficiency results in an impaired formation of RAD51 (FANCR), BRCA1 (FANCS) and ubiquitinated FANCD2 foci following MMC treatment [30] These proteins are required for homologous recombination mediated repair of double strand breaks arising during the course of ICL repair. Consistently, the homologous repair of DSBs in hSNM1B/Apollo-depleted cells was shown to be impaired, and this deficit was not increased by the co-depletion of FANCD2 [30].

Taken together, these results suggest that hSNM1B/Apollo plays an important role in the FA pathway of ICL repair. Perhaps its 5′ exonuclease activity allows hSNM1B/Apollo to process DNA at collapsed replication forks at ICLs after their unhooking; thereby creating single-stranded regions to which FANCD2 preferentially binds [48]. Loading ubiquitinated FANCD2 to the chromatin and the subsequent recruitment of other DNA repair proteins would allow for the ICL to be repaired.

### hSNM1B/Apollo is required for the repair of stalled replication forks

Faithful replication of every cell's genetic material requires a smooth and timely progression of the replication fork through the DNA double helix. However, replication barriers such as DNA lesions, secondary DNA structures or telomeres can inhibit this progression, causing replication stress and a stalling of the replication fork. While cells have mechanisms to restart stalled forks, inadequate repair leads to a collapse of the replication machinery and induces DSBs, causing chromosome instability (reviewed e.g. in [49, 50]). Sites that are particularly prone to breakage due to replication stress are called fragile sites in eukaryotes. Replication forks that stall due to ICLs blocking their path are collapsed in a controlled fashion to allow adequate repair of the lesion. Interestingly, hSNM1B/Apollo-depleted cells display a defect in the induction of DSBs after MMC exposure, indicating inadequate fork collapse at ICLs [29]. In addition, hSNM1B/Apollo interacts with MUS81-EME1, MRE11 and FANCP/SLX4, all of which are involved the formation and processing of DSBs in response to ICLs [47, 51, 52] (reviewed e.g. in [53, 54]). Taken together, these finding suggest that hSNM1B/Apollo facilitates the repair of ICLs by mediating replication fork collapse at these lesions.

Independently of its role in the repair of ICLs, the FA pathway is essential for the stabilization of stalled replication forks and protects them from degradation (reviewed e.g. in [55, 56]). Considering hSNM1B/Apollo's link to the FA pathway, Mason et al. investigated the nuclease's role in the cellular response to replication stress. The group showed that hSNM1B/Apollo-depleted cells are hypersensitive to aphidicolin, a DNA polymerase inhibitor that causes replication stress. Furthermore, they reported that hSNM1B/Apollo depletion leads to an increase in fragile site instability in control and amphidicolin treated cells. Additionally, they showed that hSNM1B/Apollo is dispensable for recognizing a stalled replication fork and activating ATR-dependent signaling pathways, but required for recruiting the repair proteins FANCD2 and BRCA1 to the lesion. FANCD2 and BRCA1 are known to protect stalled replication forks from degradation [57, 58]. hSNM1B/Apollo's nucleolytic activity is required for their recruitment, as a mutant lacking the corresponding domain is unable to rescue the phenotype [38].

Taken together, these findings indicate that hSNM1B/Apollo is involved relieving replication stress. While the exact mechanisms remain unclear, Mason et al. suggest that hSNM1B/Apollo is likely to cooperate with other nucleases such as MRE11, MUS81-EME1 and the nuclease scaffold FANCP/SLX4 to process stalled forks. Similar to its proposed function in ICL repair, hSNM1B/Apollo might process nascent lagging DNA to generate ssDNA regions that allow loading of stabilizing proteins such as FANCD2 and BRCA1. Since hSNM1B/Apollo's nuclease activity has been shown to regulate topological stress at telomeres during replication [59] (see below), Mason and colleagues hypothesize that hSBM1B/Apollo could relieve superhelical strains caused by uncontrolled unwinding after a stalled fork [38].

### hSNM1B/Apollo's role in telomere maintenance

#### hSNM1B/Apollo is a shelterin accessory protein

While early studies of hSNM1B/Apollo's functions focused on its role in the DNA damage response, it was quickly discovered that the nuclease, like many FA proteins, is also required for telomere maintenance (reviewed e.g. in [60, 61]). Mammalian telomeres are specialized nucleoprotein complexes constituting the ends of the linear chromosomes. The DNA component consists of repetitive TTAGGG sequences that terminate in a 3′ single-stranded G-rich overhang. Except during replication, this G-rich overhang invades the double-stranded telomeric DNA and forms a structure termed the “t-loop”, which limits access to the telomere terminus. This t-loop prevents cells from recognizing the ends of their chromosomes as double-strand breaks and inappropriately processing them via DNA damage repair pathways, which would lead to cell cycle arrest due to ATM/ATR signaling, chromosome fusions due to NHEJ and sequence alterations due to HR (reviewed e.g. in [62]). T-loop formation is aided by the protein component of telomeres, with the shelterin complex being central. Six core telomere-associated proteins make up this complex: POT1, which binds to single-stranded telomeric DNA, TRF1 and TRF2, which bind to double-stranded telomeric DNA, and TIN2, TPP1, and RAP1, which interconnect the proteins (reviewed e.g. in [63–65]).

Four groups independently identified hSNM1B/Apollo as a binding partner of TRF2. This protein is required for the protection of telomeres against fusion and degradation and plays a vital role in the regulation of telomere length (reviewed e.g. in [63, 66]). Additionally, TRF2 is involved in the repair of non-telomeric DNA [67, 68]. Freibaum and Counter showed that hSNM1B/Apollo co-immunoprecipitates with TRF2, co-localizes with the shelterin protein at telomeres and is stabilized through this interaction [69, 70]. Lenain et al. discovered the hSNM1B/Apollo-TRF2 interaction in a yeast two hybrid screen and a GST pulldown assay. They also reported that TRF2 is required to localize hSNM1B/Apollo to telomeres and that hSNM1B/Apollo depletion in TRF2 compromised cells leads to severe growth defects, a high incidence of DNA damage response at telomeres and an increase in the rate of telomeric fusions, while not altering the cellular levels of TRF2 [25]. Van Overbeek and de Lange found hSNM1B/Apollo and TRF2 to interact using mass spectrometry and co-immunoprecipitation and described a particular increase in DNA damage response signals at telomeres of hSNM1B/Apollo depleted cells during S-phase [26]. Results from our laboratory identified TRF2 as an hSNM1B/Apollo binding partner in a yeast two hybrid screen, co-immunoprecipitation and co-immunofluorescence studies. We were also able to show that hSNM1B/Apollo, like TRF2, accumulates quickly after the induction of DNA breaks by laser micro-irradiation [31]. Finally, Chen and colleagues used *isothermal titration calorimetry* to study the interaction and characterized the molecular surface that allows TRF2 to bind hSNM1B/Apollo [71]. Together, these findings establish hSNM1B/Apollo as a shelterin accessory protein that contributes to the protection of telomeres during or shortly after replication, without yet elucidating the mechanisms by which it does this.

### hSNM1B/Apollo aids telomeric DNA replication by reducing topological stress

Telomeric doublets were reported by van Overbeek and de Lange after hSNM1B/Apollo depletion in human cells and could be the consequence of impaired telomeric replication [26]. Ye and colleagues investigated hSNM1B/Apollo's role in the progression of the replication fork through telomeric DNA by examining the replication of an experimentally inserted telomeric sequence at an internal site of chromosome 4, allowing to discriminate interstitial from terminal events of telomere replication. Remarkably, they discovered that the nuclease domain of hSNM1B/Apollo prevents the activation of a strong DNA damage response at the telomeric sequence. Furthermore, they reported that overexpression of hSNM1B/Apollo or TRF2 rescued telomeric deficiencies caused by the depletion of topoisomerase 2α, a protein required for relieving topological stress during DNA replication. Taken together with the group's findings demonstrating that TRF2 preferentially binds positively supercoiled DNA, a model in which TRF2 functions as a sensor of aberrant telomeric topology, recruiting and controlling proteins such as hSNM1B/Apollo to relieve topological stress was proposed [59]. This pathway may even be present throughout the genome, since TRF2's preference for positively supercoiled DNA is not limited to telomeres and, as discussed above, hSNM1B/Apollo has been implicated in the resolution of superhelical strain at stalled replication forks caused by ICLs [38]. Further studies will hopefully shed light on the exact mechanism of hSNM1B/Apollo's role in facilitating DNA and, particularly, telomeric replication.

### hSNM1B/Apollo contributes to telomeric overhang maintenance

The discovery of hSNM1B/Apollo's role in the maintenance of telomeres combined with its 5′ DNA exonuclease activity lead to speculation about the protein's role in generating the 3′ single-stranded overhangs at telomeres essential for their protection [25, 69]. The replication of lagging-strand telomeres results in a small 3′ overhang due to the removal of the final RNA primer. Leading-strand telomeres, however, are replicated in a continuous manner, leaving them blunt-ended and requiring resection by a previously unknown 5′ nuclease (reviewed e.g. in [72, 73]).

In 2010, two groups independently generated *mSnm1b/Apollo* null alleles in mice by deleting different exons and identified the nuclease's pivotal role in 5′ end resection at telomeres [74, 75]. Wu and colleagues described a moderate DNA damage response at telomeres during early to mid S phase in *mSnm1b/Apollo*^–/–^ mutants, confirming their earlier results suggesting the nuclease's involvement in telomeric protection throughout replication. They also showed that mSNM1B/Apollo's interaction with TRF2 and its nucleolytic activity are essential for preventing the observed DNA damage signals. Perhaps most strikingly, they reported increased telomeric fusions in *mSnm1b/Apollo*^–/–^ MEFs involving exclusively leading-strand telomeres. Consistent with impaired 3′ overhang generation at leading-strand telomeres, they observed 30–40% less single-stranded telomeric DNA in *mSnm1b/Apollo*^–/–^ mutants – close to the expected 50% drop in total overhangs if equal overhang lengths at leading- and lagging-strand telomeres were to be assumed [76]. Interpreting their data, they suggest a model where TRF2 recruits mSNM1B/Apollo to leading end telomeres in order to generate the 3′ overhang immediately after their replication. In a study building on these findings, the group examined the mechanisms balancing the amount of telomeric resection and showed that the shelterin component POT1b inhibits telomeric hyperresection by mSNM1B/Apollo [76]. Lam and coworkers too proposed a role for SNM1B/Apollo in 3′ overhang generation. Their study revealed a high rate of telomere fusions in *mSnm1b/Apollo* null MEFs mainly involving leading-strand telomeres and indicated that the shelterin complex TPP1-POT1 cooperates with mSNM1B/Apollo in protecting telomeres from engaging in DNA repair after their replication. They speculated that NHEJ is the pathway inducing instability in unprocessed telomeres since *Ku70* deletion rescued the telomeric phenotype of *mSnm1b/Apollo*^–/–^ mutants [75]. Both groups also addressed the question of whether SNM1B/Apollo's nuclease activity is required for its’ telomeric functions and reported different results. While two nuclease deficient mutants created by Lam et al. mimicked *mSnm1b/Apollo* null cells, one of two nuclease deficient mutants generated by Wu et al. was partly able to repress the fusion of leading-strand telomeres while still showing the phenotype of reduced single-strand telomeric DNA and S-phase specific telomeric DNA damage signals. These differences could be caused by residual nuclease activity in the examined mutant, although another intriguing explanation is that SNM1B/Apollo itself could protect leading-end telomeres against fusions. Different results regarding the effect of ATM signaling on telomeric fusions in *mSnm1b/Apollo*^–/–^ MEFs were also reported: Wu et al. stated that ATM depletion inhibits telomeric DNA damage signals in *mSnm1b/Apollo*^–/–^ MEFs, while Lam et al. showed that *mSnm1b/Apollo^–/–^ATM^–/–^* double mutants had similar rates of fused telomeres as *mSnm1b/Apollo*^–/–^ mutants alone.

Despite some conflicting results, these findings greatly expanded the understanding of SNM1B/Apollo's role in telomere maintenance and confirmed earlier speculation on the nuclease's contribution to 3′ overhang generation. *mSnm1b/Apollo*^–/–^ MEFs are viable and show some intact leading-strand telomeric overhangs, therefore other nucleases must also be involved in their maintenance or are at least able to compensate for the loss of mSNM1B/Apollo. It will be of great interest to identify these contributors and study the interplay of nucleases. Furthermore, it will be necessary to examine whether these findings can be replicated in a human model, especially because studies have shown that siRNA mediated depletion in human cells triggers telomere deprotection without loss of the 3′ overhang [26, 59]. This finding may reflect different functions of SNM1B/Apollo in mice and humans or the need to fully deplete cells of the nuclease in order to study its role in the generation of 3′ overhangs. The one human *hSNM1B/Apollo* mutant model available, however, is derived from a Hoyeraal–Hreidarsson patient and expresses a variant that is unable to bind TRF2 [77] (see below). This interaction was found to be crucial for hSNM1B/Apollo's telomeric functions [74], therefore, cells with different mutations would be required to study the nuclease's role in the protection of human telomeres.

### Protein-protein interactions involving hSNM1B/Apollo

hSNM1B/Apollo's known interacting partners are listed in Table 1 and Figure 1 and some have already been discussed. Additionally, hSNM1B/Apollo was shown to bind numerous heat shock proteins [39]. These proteins form a family of molecular chaperones and play an important role in protein homeostasis by stabilizing and activating proteins. Several reports also suggest that HSP70 proteins function in DNA repair, with depleted cells showing hypersensitivity towards IR and UVC as well as impaired CHK1 activation [78–80]. Bae and colleagues found hSNM1B/Apollo to interact with Mre11 and Rad50 [29], two proteins of the MRN complex, which plays an important role in the repair of DSBs and the activation of cell cycle checkpoints in response to IR. hSNM1B/Apollo also interacts with PSF2, which is part of the GINS complex and is essential for DNA replication [81, 82]. Interestingly, PSF2 depletion renders HeLa cells hypersensitive towards MMC treatment [83]. Perhaps the best-studied interaction of hSNM1B/Apollo is with the shelterin component TRF2, as discussed above [25, 26, 31, 69, 84]. Taken together, the majority of the binding partners identified so far belong to one or both of two groups – DNA damage response and telomere maintenance proteins, reflecting the dual function of hSNM1B/Apollo.

**Table 1:** hSNM1B/Apollo interacting proteins and the methods used for their identification

Protein	Methods	Source
TRF2	Co-localization/immunofluorescence, Co-immunoprecipitation, mass spectrometry, yeast two hybrid screen, GST pulldown assay, isothermal titration calorimetry	[25, 26, 31, 69, 71]
MRE11	Co-immunoprecipitation	[29]
RAD50	Co-immunoprecipitation	[29]
MUS81	Co-immunoprecipitation, GST pulldown assay	[29] [81]
FANCD2	Co-immunoprecipitation	[29]
hSNM1B/Apollo	Co-immunoprecipitation	[26]
HSC70	Tandem affinity purification with mass spectrometry	[39]
HSP70	Tandem affinity purification with mass spectrometry; validated in Western Blot	[39]
HSP72	Tandem affinity purification with mass spectrometry	[39]
HSP60	Tandem affinity purification with mass spectrometry; validated in Western Blot	[39]
Class II β-Tubulin	Tandem affinity purification with mass spectrometry	[39]
astrin	yeast two hybrid screen, GST pulldown assay, Co-localization/immunofluorescence	[32]
FANCP/SLX4	Co-immunoprecipitation	[47]
PSF2	yeast two hybrid screen, Co-immunoprecipitation	[81]

### SNM1B/Apollo's role in disease

#### mSnm1b/Apollo is essential for normal embryonic development in mice

Akhter et al. described the effects of *mSnm1b/Apollo in vivo* utilizing a homozygous null mouse model with exon 4 deleted. The mice died at birth with defects in multiple organ systems and severe developmental delays. The corresponding MEFs showed impaired cell proliferation due to frequent telomeric fusions. Interestingly, deficiency of Ku70, a protein essential for NHEJ, rescued the mutant phenotype. p53 deficiency, however, did not, suggesting that p53-dependent apoptosis is not responsible for the proliferation defects. Instead, these results indicate that *SNM1B/Apollo* is required to inhibit NHEJ at telomeres, thereby maintaining genomic integrity [85].

### hSNM1B/Apollo in human disease

Considering the chromosomal breakage phenotype of hSNM1B/Apollo-depleted cells and the nuclease's role in DNA replication, repair and telomeric maintenance, it seems reasonable to consider whether a null mutation could cause a chromosome instability syndrome in humans similar to Fanconi anemia, Nijmegen breakage syndrome or ataxia telangiectasia. Interestingly, no *hSNM1B/Apollo* null alleles have been identified in humans thus far and deleting the gene in mice results in perinatal lethality, suggesting that hSNM1B/Apollo might be essential for survival in humans as well. However, different variants of the gene have been identified which are associated with human disease.

### A splice variant of hSNM1B/Apollo causes Hoyeraal–Hreidarsson syndrome

Touzot et al. discovered an *hSNM1B/Apollo* splice variant in a patient with Hoyeraal–Hreidarsson syndrome, a severe form of dyskeratosis congenita characterized by bone marrow failure, immunodeficiency and cerebellar hypoplasia. This mutation results in a dominant-negative version of hSNM1B/Apollo truncated at amino acid 416 and thereby lacking the domain required for binding TRF2. Interestingly, cells derived from this patient showed no hypersensitivity towards ICL-inducing agents, but major telomeric defects in the form of accelerated telomere shortening, telomere fusions and telomeric doublets [77].

### hSNM1B/Apollo variants and cancer risk

Polymorphisms in several genes involved in cellular response to DNA damage, DNA repair and telomere maintenance are known to contribute to individual cancer risk (reviewed e.g. in [86, 87]). hSNM1B/Apollo is thought to function in all three of these processes and consequently, single nucleotide polymorphisms (SNPs) at the *hSNM1B/Apollo* locus have been included in studies evaluating the association of common SNPs in candidate genes with various types of cancer. Liang and colleagues investigated a total of 2964 tag SNPs in 131 DNA repair genes in 586 individuals (*N* = 183 diagnosed with cutaneous malignant melanoma (CMM) and *N* = 379 controls) from 53 melanoma-prone families of Caucasian origin. While variants in two genes, *POLN* and *PRKDC* were significantly associated with CMM, *hSNM1B/Apollo* variants showed suggestive association (gene specific *p* = 0.0006) with this type of melanoma after Bonferroni correction [88].

A set of gene variants in gene regions of 22 telomere structure and maintenance genes were analyzed in colorectal, breast, prostate, ovarian and lung cancer by Karami and co-workers. They analyzed 204,993 SNPs in 61,851 cancer cases and 74,457 controls of European descent in their meta-analysis and identified seven novel loci, among them the *hSNM1B/Apollo* region: rs974404 was inversely associated with prostate and lung cancers and rs12144215 was inversely associated with colorectal, breast and prostate cancers [89].

A two-step genome wide association analysis (GWAS) strategy was used by a consortium investigating breast cancer in study participants of European origin. In the first step they performed a meta-analysis including 10,052 cases and 12,575 controls. As a result, 29,807 SNPs were selected for further genotyping and meta-analysis in another 45,290 cases and 41,880 controls. The hSNM1B/Apollo gene region was among the 41 newly identified breast cancer associated loci in this study, with rs11552449, a coding hSNM1B/Apollo variant (pHis61Tyr), showing genome wide significance (*P* = 1.8 × 10^–8^) [90]. Interestingly, the very same SNP was selected for a study of known breast cancer risk SNPs associated with differential transcript isoform expression. The authors reported that rs11552449 was significantly associated with differential splicing of exon 2 and that inclusion of exon 2 was significantly associated with breast cancer. However, differential splicing of transcripts of the close neighbor gene *PHTF1* was also associated with rs11552449 in this study and it was impossible to definitively identify which of the two genes was causally associated [91].

Natrajan and colleagues fine-mapped the chromosomal breakpoints in four Wilms tumor samples, which have been previously shown to be located in a 1.78 Mb interval of chromosome 1p13. In one of the tumors the breakpoint was located in intron 3 of hSNM1B/Apollo. Analysis of the surrounding genome architecture did not reveal any sequence features (e.g. repetitive elements) that obviously relate to the origin of the aberration, suggesting that this alteration might have played a role in tumorigenesis [92].

### hSNM1B/Apollo as a possible target in cancer therapy

Considering the hypersensitivities of hSNM1B/Apollo-depleted cells towards ICL-inducing agents such as MMC or cisplatin, drugs that are routinely used in cancer therapy, it has been hypothesized that specific hSNM1B/Apollo inhibitors could be used to sensitize tumors to these substances [20]. Due to the structural similarities of hSNM1B/Apollo's active site and bacterial MBLs, candidate compounds are already available in the form of MBL inhibitors. Indeed, Lee and colleagues recently showed that cephalosporins are competitive inhibitors of hSNM1B/Apollo and hSNM1A [93]. Further studies will be needed to identify selective inhibitors to use in target validation studies.

### Concluding remarks

Genomic integrity is maintained through manifold biological processes involving DNA replication, damage signaling and DNA repair. Since the identification of its full coding sequence in 2004, studies on the function of the 5′ exonuclease hSNM1B/Apollo have revealed its involvement in both the DNA damage response and in telomere maintenance. Interestingly, many of the protein's functions are analogically exercised in both pathways. During DNA replication and ICL repair, hSNM1B/Apollo is believed to process stalled replication forks in a manner that allows for the loading and subsequent recruitment of other proteins involved in repairing the respective lesion. Similarly, hSNM1B/Apollo generates the 3′ overhang at telomeres necessary for the binding of shelterin components and thereby the protection of chromosome ends from inappropriate DNA damage processing. hSNM1B/Apollo's nuclease activity has furthermore been suggested to relieve topological stress both at stalled forks and during telomere replication. The involvement of the nuclease in these pathways is in complete accordance with the reports of polymorphisms within its gene associated with various types of cancers. Altogether, studies examining the diverse functions of hSNM1B/Apollo have greatly increased and will continue to expand our understanding of cellular mechanisms ensuring genomic integrity.
